# Digital or Digitally Delivered Responses to Domestic and Intimate Partner Violence During COVID-19

**DOI:** 10.2196/19831

**Published:** 2020-07-30

**Authors:** Chuka Emezue

**Affiliations:** 1 Sinclair School of Nursing University of Missouri-Columbia Columbia, MO United States

**Keywords:** COVID-19, pandemic, mental health, digital interventions, technology, coronavirus, domestic violence, prevention, abuse, intimate partner violence

## Abstract

Before the coronavirus disease (COVID-19), 1 in 3 women and girls, globally, were victimized by an abusive partner in intimate relationships. However, the current pandemic has amplified cases of domestic violence (DV) against women and girls, with up to thrice the prevalence in DV cases compared to the same time last year. Evidence of the adverse effects of the pandemic on DV is still emerging, even as violence prevention strategies are iteratively being refined by service providers, advocacy agencies, and survivors to meet stay-at-home mandates. Emotional and material support for survivors is a critical resource increasingly delivered using digital and technology-based modalities, which offer several advantages and challenges. This paper rapidly describes current DV mitigation approaches using digital solutions, signaling emerging best practices to support survivors, their children, and abusers during stay-at-home advisories. Some examples of technology-based strategies and solutions are presented. An immediate priority is mapping out current digital solutions in response to COVID-19–related DV and outlining issues with uptake, coverage, and meaningful use of digital solutions.

## Domestic Violence and COVID-19

Violence against women and girls remains a social justice, human rights, and public health issue. Domestic violence during the coronavirus disease (COVID-19) pandemic has been aptly described as a “shadow pandemic,” by the Executive Director of United Nations (UN) Women. This is in light of local and global emerging statistics that show exponential spikes in domestic violence (DV) incidents (compared with the same period last year) [1-5]. From high-tech cities to low-tech rural areas across the world, there have been noted COVID-19–related upsurges in crisis calls to law enforcement and DV hotlines since the first week of the lockdowns [1-5]. The usual channels of support are now jeopardized by stay-at-home and social distancing mandates, so DV victims, many of whom are sequestered with their abusers, must find alternative means of support and safety, hence the focus on digital and technology-based DV mitigation strategies.

Accordingly, this perspective piece rapidly reviews evolving digital responses to DV in the wake of the COVID-19 pandemic. Specifically, this paper describes emerging best practices to support survivors, their children, and even abusive partners during stay-at-home advisories. Some examples of technology-based strategies and solutions are presented, and the paper concludes with emergent priorities including the need to map out current digital solutions in response to COVID-19–related DV, even as we outline new and old issues with the uptake, scope, and meaningful use of these digital solutions.

Before COVID-19, 1 in 3 (or 243 million) women and girls, globally, experienced violence by an abusive partner in intimate and casual relationships [6,7]. However, this pandemic has amplified domestic and intimate partner violence against women and girls, with increasing spates of physical, psychological, and sexual violence, and other co-occurring violence typologies (eg, child abuse, elder abuse, pet abuse, femicide, cyberviolence, stalking, and financial abuse) [8-10]. Besides, a current proliferation of gun and ammunition sales as families brace for COVID-19–related uncertainties have led to worrying fears of increased femicide (the intentional murder of female partners) since lockdown mandates were established [2,3]. Newer forms of partner abuse have also emerged, including reports of violent abusers threatening to infect a partner or their children in the home with the coronavirus.

No doubt, although DV survivors and victims are now dangerously sequestered with an abusive partner and are enduring adverse physical, psychological, social, and economic conditions [11-15], emergent mental health outcomes will be exacerbated by lockdown mandates. Current social distancing and stay-at-home mandates will also amplify pre-existing depression, anxiety disorders, suicidal ideation [12,15,16], panic disorders, posttraumatic stress disorder [17], as well as other mental and psychosomatic distress reactions (eg, insomnia, hyperarousal, avoidance, numbing, obsessive-compulsive disorder, and personality disorders) [18]. Within communities, epidemiologic evidence shows an intensification (ie, increased prevalence and severity) of other forms of gender-based violence including rape, sex trafficking, female genital mutilation or cutting, and early or child marriages during and immediately after catastrophic events of this magnitude [2,3,9,19-22]. The usual support networks for survivors have been compromised, as DV service providers contend with new and extraordinary challenges related to this pandemic. This disruption in the delivery of essential services has prompted growing calls for evidence-based, free, low-burden, and scalable digital solutions that reach survivors where they are in response to the rising shadow pandemic of DV and its projected residual effects.

## Prepandemic Digital Interventions for Domestic and Intimate Partner Violence

### Current Digital Interventions

Before this pandemic, the delivery of digital health interventions via mobile devices (mobile health), web-based, and electronic health platforms (such as online and social media modalities) had become prominent. Likewise, with those who experience DV, there is growing evidence of the acceptability and feasibility of trauma-informed digital or digitally delivered interventions that prevent violence, increase the safety and decision-making of persons in an abusive relationship, and ultimately link them to trusted support [23-27]. Evidence shows some DV survivors prefer the practicality and confidentiality of technology-enabled interventions and guided online support (as opposed to in-person face-to-face services such as group counseling and individual therapy), making this a highly acceptable form of intervention delivery [23-28]. To their merit, technology-based interventions prioritize survivor privacy and safety, and offer personalized real-time access to DV screening, risk awareness, and support services [24,25]. Substantively, digital interventions provide safer options for leaving or navigating an abusive relationship (ie, safety planning). These interventions also offer risk and danger assessment, psychoeducation, referral to trusted care, and can be tailored to unique social ecologies in ways that mitigate user burden and maximize safety [25]. In this COVID-19 era, these digital interventions have become of value to socially and physically isolated people who experience abuse, especially while survivors are sequestered at home with abusive partners.

Examples of pre–COVID-19 evidence-based digital interventions and safety decision aids include the myPlan app [25,29], I-DECIDE [30], and *iSafe* [26]. These web- and app-based digital tools are free and easy to access, and have been tested for efficacy and effectiveness (using randomized controlled trials), and with several survivor cohorts. These apps have been used successfully with some Indigenous; immigrant; same-sex; lesbian, gay, bisexual, transgender, and queer+; college; pregnant; and rural female survivors [25-27,31-33].

### How They Work: Digital Interventions for Domestic and Intimate Partner Violence

As an example, the myPlan app [34]—first designed as a computer-based intervention—serves primarily as a decision aid to help survivors make informed decisions about their safety and well-being. Leveraging a strength-based and empowerment-focused approach (ie, Dutton’s empowerment model), the authors of the app suggest it increases the survivor’s autonomy and agency. Specifically, the myPlan app—similar to most DV digital interventions—seeks to educate survivors on relationship red flags and fatality risk using a danger assessment component [35]. The myPlan app also estimates survivor priorities for safety, creates a checklist of survivor-specific safety behaviors, and designs a tailored safety plan based on the survivor’s level of danger and achievable safety behaviors [25]. The goal is to connect survivors to meaningful support as they see fit. A 12-month follow-up of a US study showed the myPlan app reduced total decisional conflict (*P*=.01), increased feelings of being supported in deciding what to do in an abusive relationship (*P*=.01), and increased the likelihood of creating a safety plan [25]. Similarly, a New Zealand study using the web-based *iSafe* intervention with Māori Indigenous women showed a reduction in violence exposure for Māori survivors (adjusted intervention estimate −14.19; 95% CI −24 to −4.37) at 6 months and at 12 months (adjusted intervention estimate −12.44; 95% CI −23.35 to −1.54) compared to non-Māori survivors (adjusted intervention estimate 0.76; 95% CI −5.57 to 7.09) in the same period. The *iSafe* intervention also reduced depression for Māori survivors at 3 months only (adjusted intervention estimate −7.75; 95% CI −15.57 to 0.07) compared to non-Māori survivors (adjusted intervention estimate 1.36; 95% CI −3.16 to 5.88) [26]. Building on this efficacy, several country-specific adaptations and clinical trials are now in progress. Qualitative studies also show wide acceptance and satisfaction with these digital tools by survivors [25,30].

These digital interventions are of benefit now more than ever, as they support hard-to-reach low-income survivors of partner violence [33], especially in health provider shortage areas, where victimization may intersect with other determinants of violence [24,25,29,36]. However, these digital interventions preclude the unique circumstances of a global pandemic.

## Current Technology-Based Strategies

### Remotely Working With Survivors

According to a report by UN Women [10], free, round-the-clock digital solutions such as 24/7 hotlines have become a treasured resource during the lockdowns. Consequently, several countries have expanded online web-based services for victims of violence, with 24/7 digitalized responses prioritizing the uniqueness of social and physical isolation [37]. Specific digital responses include the use of DV hotlines, web-services (eg, tele-counseling and telepsychiatry), and a growing corpus of recommendations to guide the selection of prepandemic proprietary smartphone apps. Issues like user safety, user burden, gender digital divides, data privacy, and confidentiality have become paramount priorities and challenges of digital DV intervention. Digital solutions now attempt to augment but not compete with nondigital traditional services [23-28,38]. However, they pose complex challenges as digital responses strive to be convenient but inconspicuous, given that abusers are likely to intercept them in close quarters, further compromising the safety of survivors and their children. At a macrolevel, there have been several published tech safety guidelines by mainstream DV agencies, including the National Network to End Domestic Violence (NNEDV), the National Coalition Against Domestic Violence, the National Domestic Violence Hotline, the Sexual Violence Research Initiative (SVRI), and the Center for Court Innovation. These tech safety guidelines have become useful evaluative tools as survivors, service providers, bystanders, and advocates decide on which digital solution is best for whom, signaling emerging best practices.

In low- and middle-income settings, there are media reports of pragmatic support services delivered via low-data messaging platforms (eg, WhatsApp, WeChat, Sina Weibo) with on- and offline capabilities. Specifically, DV agencies use these platforms to send and receive confidential information during client check-ins and meetings. Besides, online support groups provide a platform for survivors to disclose and document their abuse. These social media platforms also help survivors participate in asynchronous real-time chat forums and virtual meetups with other survivors. With concerns for safety, several guidelines are in place to ensure these online spaces remain confidential, private, and secure. For example, the TechSafety webpage of NNEDV provides several comprehensive guidelines for online, social media, device, and browser safety.

Since awareness is a key prevention strategy, social media users around the world are showing solidarity with survivors by using hashtags to call attention to COVID-19–related spikes in DV. A precursory infoveillance of internet and social media ecosystems using basic data extraction methods (eg, Twitter mining, web searching) revealed hashtags referencing the rise of DV since January 2020. In addition to other trending hashtags (#FlattenTheCurve, #StayHomeSaveLives), DV hashtags like #YouAreNotAlone (launched as an official campaign hashtag by the UK government) and #AntiDomesticViolenceDuringEpidemic (searched more than 3000 times on the Weibo app alone) have become essential tools of digital protest, social activism, and DV consciousness-raising on social media [4,39].

These digital efforts also include men and boys who are abusive or at-risk for becoming abusers. DV social media campaigns (such as the MenEngage campaign) target men and fathers at home, stressing the benefits of healthy relationships, role modeling, and positive masculinity. Long before the pandemic, the MenEngage campaign has been a popular international men’s program with over 700 nongovernment organizations and country partners all over the world. However, DV spikes have underscored this as a crucial digital strategy among men and boys. This tactic is critically important, as current spates of furloughs and job losses are predictive of economic stressors, which can lead to feelings of helplessness, anger, worries of infection, and emotional dysregulation—all likely to increase the frequency, volatility, and severity of DV among families with an abuser already present [3,4].

### Remotely Working With Perpetrators and Abusers

Family court and Family Justice Center proceedings have also ground to a halt, slowing down filing and sentencing procedures. This is a crucial impediment, as orders of protection through the family court are a vital resource for survivors seeking to hold their abusers accountable, especially those in underserved areas [40,41]. Court systems experiencing delays in usual court processes (physical appearances at court, processing bail, bonds, and warrants) are now fast-tracking lockdown-related case management by switching to digital and virtual procedures (eg, remote hearings). Some courts are putting aside “non-urgent court matters” for the groundswell of DV-related cases [42]. Continuing Operations Plans from court systems have been amended to ensure the uninterrupted continuance of court events to allow for virtual and remote procedures. Some digital responses include implementing electronic monitoring of bail and sentencing procedures, keeping and tracking attendance records using digital devices, court appearances and child custody hearings via telepresence, and digital filing of restraining orders. Other pragmatic solutions include extending the duration for restraining orders to assure the protection of survivors; providing extra notice of hearings; making backup plans for technology- and internet-impaired clients; training judges, attorneys, and court staff; using video conferenced interpreters; and publishing best practices and how-tos for remote hearings [4,37]. For example, some courts in California now use emergency civil orders of protection requested via drop box, online request forms, email, and fax, issued for 30 days any time of the day. However, court systems that are not already technology-enabled (with audiovisual, text, screen-sharing, and file transfer functions) may find it challenging to pivot to digital methods. Besides, court systems may find it challenging to secure funding to procure telecommunication equipment to facilitate remote court proceedings [43]. This pandemic has highlighted the usefulness of digital interventions with abusers, however, these digital strategies are not without issues, as security, privacy, and access problems remain prevalent.

Batterer rehabilitation programs have always offered online classes to deliver program curricula to abusers. However, rehabilitation programs may now fully use digital modalities for remote offender counseling, group or individual sessions, intake processing for new abusers, and monitoring completion of assignments as part of their rehabilitation program. Digital modalities may also be of use for other urgent legal proceedings such as filing disputes for divorce and child custody. In some areas, parole officers are encouraged to use digital tactics such as on-site but socially distanced phone calls to parolees to minimize exposure to the coronavirus. Of note, although vital to abuser accountability, it is likely these digital solutions may compromise effective monitoring of DV and fail to detect recidivist behaviors with abusers [43]. Another way that technology is used is with the early and supervised release programs of low-level, aging, pregnant, and at-risk offenders to curtail the spread of COVID-19 in prisons and jails. Victim notification apps such as VINEmobile [44] are now being leveraged to notify survivors of changes in their abuser’s custody status, case details, arrests, bond hearings, and other legal activities.

## Others Responding to COVID-19–Related Domestic Violence

The NNEDV published a Digital Services Toolkit in response to COVID-19 [45]. Some topics covered include “Using Technology to Communicate with Survivors During a Public Health Crisis,” and “Step-by-Step Guide to Choosing Tools for Digital Services” [45]. Service providers, advocates, and clinicians can use these digital compendia as a checklist to gauge the credibility, usefulness, and safety of digital interventions for survivors. Similar best practices for technology use are being used around the world. For example, in Beijing, China, the Yuanzhong Family and Community Development Service Center published an online legal aid of special manuals (translatable to English) for survivors and service providers [39]. The American Psychiatric Association’s “*Hierarchical framework for evaluation and informed decision-making regarding smartphone apps for clinical care*” is also a suggested checklist for checking the credibility of digital interventions that specifically target mental health outcomes [46].

Information on how to continue research with DV survivors in light of COVID-19 is also emerging. On the one hand, data collection to support vulnerable survivors is critical information; however, data collection is threatened by the heightened risk of doing so with the added insecurities brought on by COVID-19. Considering this conundrum, Elizabeth Dartnall (Executive Director of the SVRI) and Ellen Bates-Jefferys (Senior Research Associate at Innovations for Poverty Action) have recommended tools for remotely gathering research data during the lockdown [47]. Some strategies involve replacing face-to-face data gathering methods with computer- and mobile phone–assisted surveys, websites such as SurveyMonkey [48], and instant messaging platforms such as WhatsApp. This comes as DV researchers are forced to change their research and survey protocols by switching to phone call protocols and online consenting processes using enhanced data management plans after research review board amendments [47]. Although it is crucial to understand the scope of COVID-19–related DV, data collectors raise essential concerns about ethicality, data quality, survivor safety, retraumatization, confidentiality, and data ownership during this period [47].

Spurred by DV advocate organizations and human rights activists, service providers are recommending digital platforms to reach *all* survivors. For instance, the National Domestic Violence Hotline and the National Teen Dating Abuse Helpline continue to publish their 1-800-799-SAFE (7233) and 1-866-331-9474 numbers, respectively, offering free and real-time talk and chat services in English and Spanish. Similar local and global efforts have inspired the innovative use of emergency websites and crisis numbers responsive to sexual and gender minority groups, including male survivors—who also face violence from male or female abusers. For instance, Futures Without Violence published a list that includes the Trans Lifeline (1-877-565-8860) staffed by trans facilitators for trans and questioning folks, as well as the Deaf Hotline—an around-the-clock video phone (1-855-812-1001), email, and chat service for deaf and hard of hearing survivors. At the grassroots level, online antiviolence coalition-building, social media consciousness-raising, online crowdfunding, electronic filing services for court services and proceedings have become welcome digital strategies to support survivors, prevent abuse, and even hold abusers accountable.

At the governmental level, key leaders and heads of government met at the Women Leaders’ Virtual Roundtable on COVID-19 to re-emphasize the short- and long-term detrimental effect of the COVID-19 pandemic on women and girls. Findings from this meeting identified priority policy measures to facilitate “a more gender-inclusive recovery path” [10]. Governments are advised to use coordinated multi-sector community-led responses to exempt survivors from shelter-in-place orders and to sustain much-needed funding to key agencies. Other recommendations include efforts to classify DV shelters as essential services and increase necessary resources to DV and allied services for gender-diverse victim groups, including trans men and women who face exponential levels of partner or nonpartner violence.

## Challenges With Using Technology

However, novel digital modalities are not without their shortcomings. For example, survivors (and even abusers in treatment) may face inherent structural and practical barriers to accessing digitalized services while sheltered in-place. Specific challenges may include internet connectivity issues (in low- or dead-zone internet coverage areas) and in no-tech and low-tech situations, leading to high-data burden and accessibility issues. These barriers can significantly impair help-seeking and are pronounced in unincorporated rural communities, among low-income users, and among older adult users (so-called “digital immigrants”) who may be unfamiliar with new technologies. Survivors also worry about their rights and choices when using impersonal digital technologies to discuss such a sensitive and dangerous issue [24,26,38]. In low- and middle-income countries, reduced use of counseling services by phone, SMS, and email is linked to profound gender digital divides, technical illiteracy, and device disparities, making digital resources supplementary at best [10,49]. Not to mention the challenges service providers face in meeting the cultural needs of some vulnerable cohorts (eg, immigrant and minority groups) [50].

In addition, there are practical barriers in the home. Abusers are known to use a recipe of digital trackers, GPS, and spyware to covertly and overtly monitor the online presence of the person they are abusing to maintain coercive and even deadly control [51]. Abusers may impersonate the person they are abusing and gain entrée into what is supposed to be a safe space, particularly online fora, using fake social media accounts, and under false pretexts, armed with intimate details of the person they abuse [51]. In the wake of this pandemic, emerging forms of technology-based abuse have also spiked. These include online stalking, zoombombing, cyberbullying, doxing (disclosing personal information online in retaliation), sexualized trolling, nonconsensual pornography (or revenge porn), and coercive behaviors with adverse implications for victims of online abuse, including children and adolescents [49]. In addition, stay-at-home directives will facilitate the interception and strict round-the-clock surveillance of social media and mobile devices by abusers. This will further limit known and free avenues for help-seeking and abuse disclosure [2].

To reduce this type of online abuse and surveillance, Freed et al [51] recommends that app designers and vendors set up interface-level security measures that can distinguish the abuser from the victim based on behavioral, keystroke, or contextual cues. They also recommend covert authentication and verification protocols (eg, emergency exit buttons, app lockdown, or data dump after failed password entry) integrated into DV apps and websites. Other strategies include passcodes for mobile apps, one-click access to DV hotlines, and the use of evidence-based and tailored content for unique users. As a preemptive measure, Eterovic-Soric and colleagues [52] suggest some antiviolence technologies can be used against stalkers. These include specialized stalker detection software, Tor anonymity network set-up for private online communication, and device encryption [52]. On the TechSafety webpage, NNEDV provides comprehensive pros and cons reviews on some of these apps [45]. In partnership with Facebook, NNEDV has also published a resource on “Tips for Helping a Friend Experiencing Domestic Abuse During COVID-19,” along with other COVID-19–specific guidelines for survivors, friends and family of survivors, and service providers [53].

## Challenges for DV Agencies

At the agency level, there are noted barriers to the uptake of digital solutions during social distancing and lockdown restrictions, including the burden on agency staff to appraise and become familiar with the safe use of new technologies. Nonprofit agencies on shoestring budgets also contend with overextended bandwidth, device or subscription requirements, information technology (IT) troubleshooting issues, data privacy, and data mining worries.

Even as DV agencies grapple with the learning curve of digital interventions, DV shelters expectedly get more shelter-seekers during natural disasters [9,19,21]. With COVID-19, shelters are surpassing their maximum capacity, prompting alternative sheltering options such as Hotel Assistance Programs using vacant hotel rooms and dormitories as makeshift shelters for survivors. In past disasters, survivors (mothers and children) have been turned away [19]. Still, this pandemic poses a unique complexity as DV shelters are doubly burdened with monitoring clients for symptoms of the coronavirus to enact infection control protocols.

Nonprofit DV service providers contend with funding cuts such as grant matching stipulations; overburdened services; even as their staff are exposed to their own violence online, in person, and within antiviolence spaces. Overall, funding cuts are heightened now, as cuts in government relief funding are diverted to meet other emergent needs. Fortunately, the Coronavirus Aide, Relief and Economic Security (or CARES Act) provided $45 million for programs under the Family Violence Prevention and Services Act that offers DV survivors emergency housing and other critical services during this time. The CARES Act also provides $2 million for the National Domestic Violence Hotline—a much needed digital resource. However, additional support is still needed for sexual assault programs, even as shelter and service providers continue to contend with upsurges.

In light of these, DV agency staff working under these militating conditions also face adverse psychological stress, compassion fatigue, and burnout, and may need to be intentional in acts of self-care and separation from work to continue contributing their expertise and emotional labor in support of survivors. This process is contingent on unimpeded material and moral support from us all, as supporting DV survivors and DV-impacted families is a responsibility not limited to the government, agency heads, and advocates.

## Conclusion: What Now?

Going forward, Fisher [20] emphasizes prioritizing survivor voices, rights, and perspectives in the design of trauma-informed digital intervention. Digital intervention planners are advised to use “gender mainstreaming,” feminist, and socio-ecological lenses as guiding praxis for pre- and postdisaster preparedness to respond to DV and its intersectional issues [54]. A specific example may be the use of gender-disaggregated data to understand the gendered effects of DV during and after a disaster. Emerging research must focus on understanding the residual impact of the coronavirus pandemic on family functioning in the context of DV, with specific emphasis on protective factors, resiliency, resistance, coping, and safety. Knowing the importance and value of digital interventions, DV researchers and advocates must quickly form cross-collaborations with other professionals such as app developers, using tested coordinated community networks to capitalize on current research infrastructure and expertise [55]. Peterman et al [33] recommend other strategies including training health care providers to better identify and use DV digital tools, and reinforcing digital safety nets for survivors and service providers, including digitized cash transfers, digital resources listing employment benefits, legal, health, childcare, shelter or transitional housing, and trusted psychosocial services for survivors and their families.

DV researchers must also build emergency preparedness into future service delivery protocols, building on lessons learned so far. Interventionists and disaster planners are now tasked with fully understanding the psychological consequences of social isolation on survivors and the abuse tactics of perpetrators in social isolation, and developing urgent strategies in creating, testing, and mapping out digital and digitally delivered responses for our complex digital ecosphere*.* As the tech sector continues to innovate digital tools and the government continues to facilitate the accelerated IT modernization of our digital infrastructure, partnerships between researchers, advocates, survivors, and community-based organizations have become pertinent. These collaboratives can help improve policies, regulatory mechanisms, and funding systems that advance the design, testing, and upscaling of digital interventions in DV spaces while anticipating the unintended effects these interventions may have on the health and well-being of survivors and their families. These public-private partnerships are necessary to invest in critical digital tools that streamline access to evidence-based but pragmatic digitalized services.

Last, per the gender and disaster literature, the outcomes of natural disasters are highly gendered. This disproportionately impacts women and girls by increasing their invisibility and vulnerability to gender-based forms of violence—much unlike men and boys [20]. Although the effects of disasters are felt more so at the individual level, social and economic recovery efforts will depend primarily on pre-existing socio-ecological systems and systemic vulnerabilities among at-risk groups [19,20]. These vulnerabilities are intersectional and should form the basis of disaster and vulnerability praxis, as we continue to design and fine-tune digital and digitally delivered responses to DV during disruptive events such as pandemics.

## Abbreviations

CARES: Coronavirus Aide, Relief and Economic Security
COVID-19: coronavirus disease
DV: domestic violence
IT: information technology
NNEDV: National Network to End Domestic Violence
SVRI: Sexual Violence Research Initiative
UN: United Nations
